# Crizotinib efficacy in advanced non-squamous NSCLC patients with ALK or ROS1 rearrangement

**DOI:** 10.1038/s41598-021-00309-3

**Published:** 2021-10-22

**Authors:** Paweł Krawczyk, Anna Grenda, Paulina Terlecka, Justyna Błach, Kamila Wojas-Krawczyk, Tomasz Kucharczyk, Izabela Chmielewska, Robert Kieszko, Bożena Jarosz, Michał Gil, Katarzyna Reszka, Janusz Milanowski

**Affiliations:** 1 Department of Pneumonology, Oncology and Allergology, Medical University of Lublin, Jaczewskiego 8, 20-954 Lublin, Poland; 2 Department of Endocrinology, Diabetology and Metabolic Diseases, Medical University of Lublin, Lublin, Poland; 3 Department of Clinical Immunology, Medical University of Lublin, Lublin, Poland; 4 Neuropathology Laboratory, Department of Neurosurgery and Pediatric Neurosurgery, Medical University of Lublin, Lublin, Poland

**Keywords:** Biomarkers, Diseases, Medical research, Molecular medicine, Oncology

## Abstract

In patients with advanced non-small cell lung cancer (NSCLC), comprehensive genetic diagnostics is currently carried out in order to qualify for molecularly targeted therapies and immunotherapy. The aim of the study was to assess the usefulness of the reverse transcriptase (RT-PCR) method in the diagnosis of gene rearrangements, the effectiveness of EGFR, ALK, ROS1, and PD-L1 inhibitors in first-line treatment in NSCLC patients. We enrolled 95 non-squamous NSCLC patients with known status of *EGFR*, *ALK*, *ROS1*, *MET* and *RET* genes and PD-L1 protein expression. We used the real time PCR, fluorescence in situ hybridization (FISH), immunohistochemistry (IHC) and RT-PCR techniques for determination of predictive factors. In patients with *ALK* and *ROS1* genes alteration, the median overall survival was 34 months in crizotinib treated patients and 6 months in patients who received chemotherapy (HR = 0.266, p = 0.0056). The risk of death was lower in patients treated with molecularly targeted therapies or immunotherapy compared to patients with predictive factors without personalized treatment (HR = 0.265, 95% CI 0.116–0.606) and to patient without predictive factors who received chemotherapy (HR = 0.42, 95% CI 0.162–1.09). Diagnosis of predictive factors and implementation of personalized treatment are key to prolonging the survival in advanced NSCLC patients.

## Introduction

The most common first-line personalized treatment methods available until recently in locally advanced or advanced NSCLC patients were molecularly targeted therapies for patients with *EGFR* (Epidermal Growth Factor Receptor) gene mutations, *ALK* (Anaplastic Lymphoma Kinase) and *ROS1* genes rearrangements as well as immunotherapy with anti-PD-1 (Programmed Death 1) or anti-PD-L1 (Programmed Death Ligand 1) monoclonal antibodies. Recently, new therapeutic options have emerged: inhibitors of MET (Hepatocyte Growth Factor Receptor, HGFR), RET, NTRK (Neurotrophic Tyrosine Receptor Kinase), BRAF kinases and combination therapies with the use of immunotherapy. New generations of tyrosine kinase inhibitors (TKIs) of EGFR, ALK and ROS1 have also been developed^1^.

Genetic alterations predisposing to molecularly targeted therapies occur in NSCLC patients with low frequency. In Caucasian patients with non-squamous NSCLC, mutations in the *EGFR* gene are found in 10–15% of cases, *ALK* gene rearrangement—in 4.5–7.5% of cases, *ROS1* gene rearrangement—in 1.5–2% of cases. Each of the remaining genetic abnormalities occur in 1–2% of patients. However, summing up the prevalence of the discussed group of predictive factors, even 60–70% of patients may have a genetic predisposition to molecularly targeted therapies. PD-L1 expression on ≥ 50% of tumor cells occurs in about 25% of NSCLC patients, regardless of pathomorphological diagnosis. The presence of this predictive factor qualify NSCLC patients to first-line therapy with the use of pembrolizumab or atezolizumab (recently pembrolizumab in monotherapy can also be used in patients with PD-L1 expression on ≥ 1% of tumor cells). Pembrolizumab was also effective in patients with high tumor mutation burden (TMB) and microstalite instability (MSI)^2–4^.

In-depth study of genetic abnormalities in many genes has become possible in the routine diagnosis of NSCLC patients thanks to the introduction of next-generation sequencing (NGS) technology. However, methods for fast and simple assessment of single changes in selected genes are still used. For this purpose, the real-time PCR method is used in the diagnosis of mutations in *EGFR* gene, as well as the fluorescence in situ hybridization (FISH) and immunohistochemistry (IHC) methods—in the diagnosis of abnormalities of *ALK* and *ROS1* genes. The use of reverse transcriptase PCR (RT-PCR) in the diagnosis of genes rearrangements is controversial, due to insufficient sensitivity and non-diagnostic reports resulting from mRNA degradation^1,3^.

Crizotinib is an anti-cancer drug acting as an ALK, ROS1 and MET tyrosine kinase inhibitor. Crizotinib is the first generation of ALK and ROS1 inhibitor and has been used in the treatment of locally advanced or metastatic NSCLC patients with *ALK* or *ROS1* genes rearrangements in the first-line therapy or after chemotherapy failure. The PROFILE 1007 trial was the first randomized phase III trial for NSCLC patients with *ALK* rearrangements previously treated with chemotherapy. Patients were randomized to crizotinib or chemotherapy (pemetrexed or docetaxel). Overall response rate (ORR) for crizotinib-treated patients was 65% compared to 20% in the comparator arm. The median progression free survival (PFS) was prolonged from 3 months in chemotherapy group to 7.7 months in patients treated with crizotinib^5^. The efficacy and safety of crizotinib for the treatment of patients metastatic NSCLC with *ALK* rearrangements, who had not received previous systemic treatment, were demonstrated in a global, randomized, open-label PROFILE 1014 study. Crizotinib significantly prolonged PFS compared to chemotherapy (10.9 vs 7 months) and increased overall response rate (74% vs 45%). The PFS benefit of crizotinib was consistent across subgroups of baseline patient characteristics such as age, gender, race, smoking class, time since diagnosis, performance status, and presence of brain metastases^6^. The use of crizotinib in the treatment of advanced NSCLC patients with *ROS1* rearrangement was investigated in multicenter, multinational, single-arm PROFILE 1001 study. In this study, ORR was 72% and median PFS was 19.3 months. The median overall survival (OS) at the time of data cutoff was 51.4 months^7^. In the same study, objective responses were observed in 32% of NSCLC patients with splice-site region mutations in *MET* gene (skipping mutation in exon 14). The median PFS in such patients was 7.3 months. Similar results were obtained in the AcSe study (ORR—40%, median PFS—3.6 months)^8^. These data indicated limited effectiveness of crizotinib in NSCLC patients with mutations in *MET* gene.

The goal of our research was to evaluate the usefulness of the RT-PCR method in the diagnosis of *ALK* and *ROS1* gene rearrangements. In addition, we examined the effectiveness of crizotinib compared to chemotherapy in first-line treatment in NSCLC patients with *ALK* and *ROS1* genes rearrangement. We have shown that the use of molecularly targeted therapy or immunotherapy in the predisposed patients is the only way to extend their life.

## Results

### Sensitivity and specificity of RT-qPCR test in ALK and ROS1 examination

RT-qPCR test revealed rearrangements of the *ROS1* gene in 9 patients, rearrangements of the ALK gene in 19 patients, skipping mutations in exon 14 of the *MET* gene in 5 patients and rearrangement of the *RET* gene in 1 patient. The mutation in the *MET* gene was retested and confirmed only in two patients by the next generation sequencing method (Oncomine panel on the S5 Ion Torrent platform, Thermo Fisher Scientific, USA).

Among 9 samples with *ROS1* gene rearrangements detected by FISH method, 6 samples also showed rearrangements of this gene in the RT-qPCR method (Table 1). The sensitivity and specificity of RT-qPCR method compared to FISH methods were 66.6% and 97%. The positive predictive value (PPV) and negative predictive value (NPV) of RT-qPCR method in the diagnosis of *ROS1* abnormalities was 0.67 and 0.96.

**Table 1:** Comparison of results of RT-qPCR and FISH methods used in assessment of *ROS1* rearrangements.

	Number of cases	
	RT PCR positive	RT PCR negative
FISH positive	6	3
FISH negative	3	86

Of 23 samples with *ALK* abnormalities detected by IHC and FISH methods, 16 were also positive in RT-qPCR method (Table 2). The sensitivity and specificity of RT-qPCR method compared to IHC and FISH methods were 69.6% and 96.2%. The PPV and NPV of RT-qPCR method in the diagnosis of *ALK* abnormalities was 0.84 and 0.915.

**Table 2:** Comparison of results of RT-qPCR and IHC/FISH methods used in assessment of *ALK* rearrangements.

	Number of cases	
	RT PCR positive	RT PCR negative
FISH and IHC positive	16	6
FISH and IHC negative	3	76

### Survival analysis of patients with ALK or ROS1 genes rearagament

**Figure 1:**
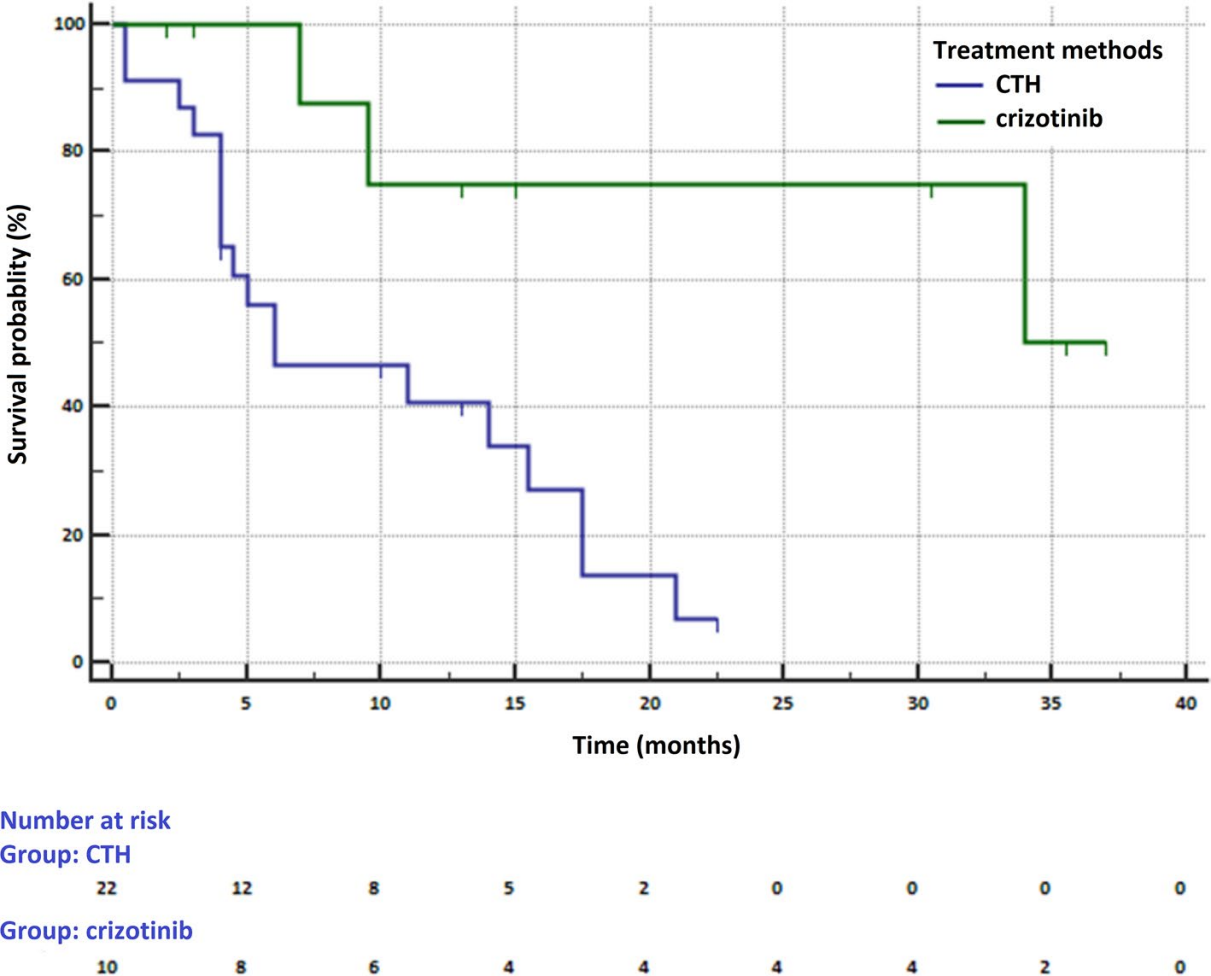
Survival of NSCLC patients with *ROS1* or *ALK* genes rearrangements who received crizotinib or chemotherapy in first-line of treatment (MedCalc Statistical Software version 18.11.6, https://www.medcalc.org).

In 32 patients with *ALK* or *ROS1* genes rearrangements, 10 patients received crizotinib and 22 patients chemotherapy in the first-line of treatment. The median overall survival was 34 months (95% CI 9.5–34 months) in crizotinib treated patients and 6 months (95% CI 4–15.5 months) in patients who received chemotherapy (HR = 0.266, 95% CI 0.104–0.678, p = 0.0056) (Fig. 1). Median PFS in this subgroup was 23 months (95% CI 6–23).

The prognosis of patients received of chemotherapy was particularly poor due to the presence of metastases to the central nervous system (CNS). In 14 (63.6%) patients receiving chemotherapy and 5 (50%) patients receiving crizotinib, CNS metastases were observed. CNS metastases occurred in 8 patients before the start of chemotherapy and in 3 patients before the administration of crizotinib. These patients were treated with CNS radiotherapy prior to first-line treatment initiation.

### Survival analysis depending on the predisposition to personalized treatment

**Figure 2:**
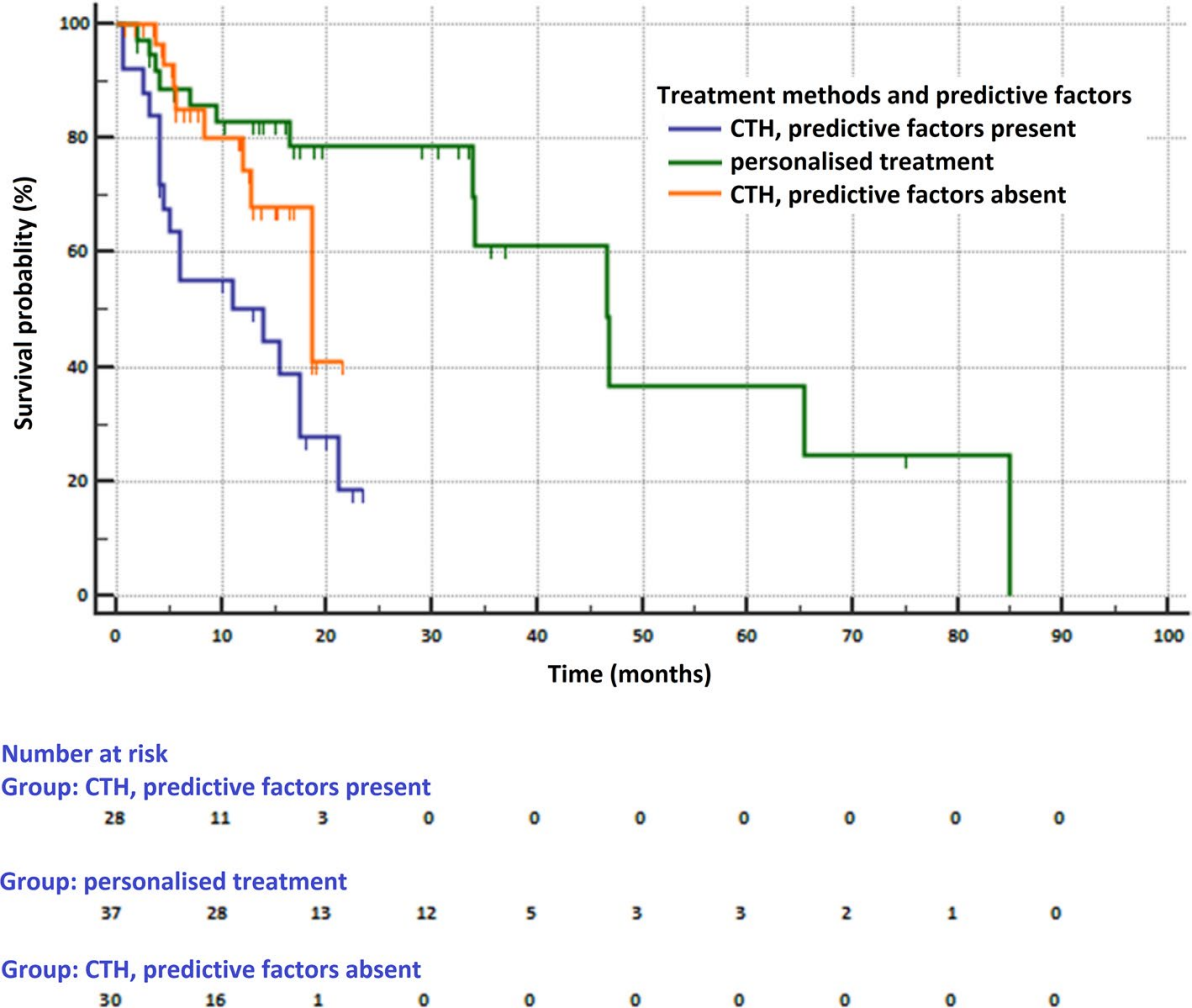
Survival of patients depending on the presence of predictive factors (mutations in the *EGFR* gene, rearrangements of *ALK* and *ROS1* genes, PD-L1 expression) and the type of treatment (MedCalc Statistical Software version 18.11.6, https://www.medcalc.org).

In total group of 95 patients, 62 patients had predisposition to first-line personalized treatment (available in Poland: tyrosine kinase inhibitors of EGFR, ALK, ROS1 or immunotherapy anti-PD-1). In this group, 20 patients received TKIs, 17 patients pembrolizumab and 34 chemotherapy. Median OS was 46.7 months (95% CI 33.9–85 months) in patients treated with TKIs or pembrolizumab (group 1), 14 months (95% CI 4.5–17.5 months) in patients with predictive factors who received chemotherapy (group 2) and 18.6 months (95% CI 12.8–18.6 months) in patients without defined predictive factors, who received chemotherapy (group 3, p = 0.0002, Fig. 2). The risk of death was lower in patients in group 1 compared to patients in groups 2 (HR = 0.265, 95% CI 0.116–0.606) and 3 (HR = 0.42, 95% CI 0.162–1.09). The risk of death was also lower in patients in group 3 compared to patients in group 2 (HR = 0.631, 95% CI 0.295–1.35).

**Figure 3:**
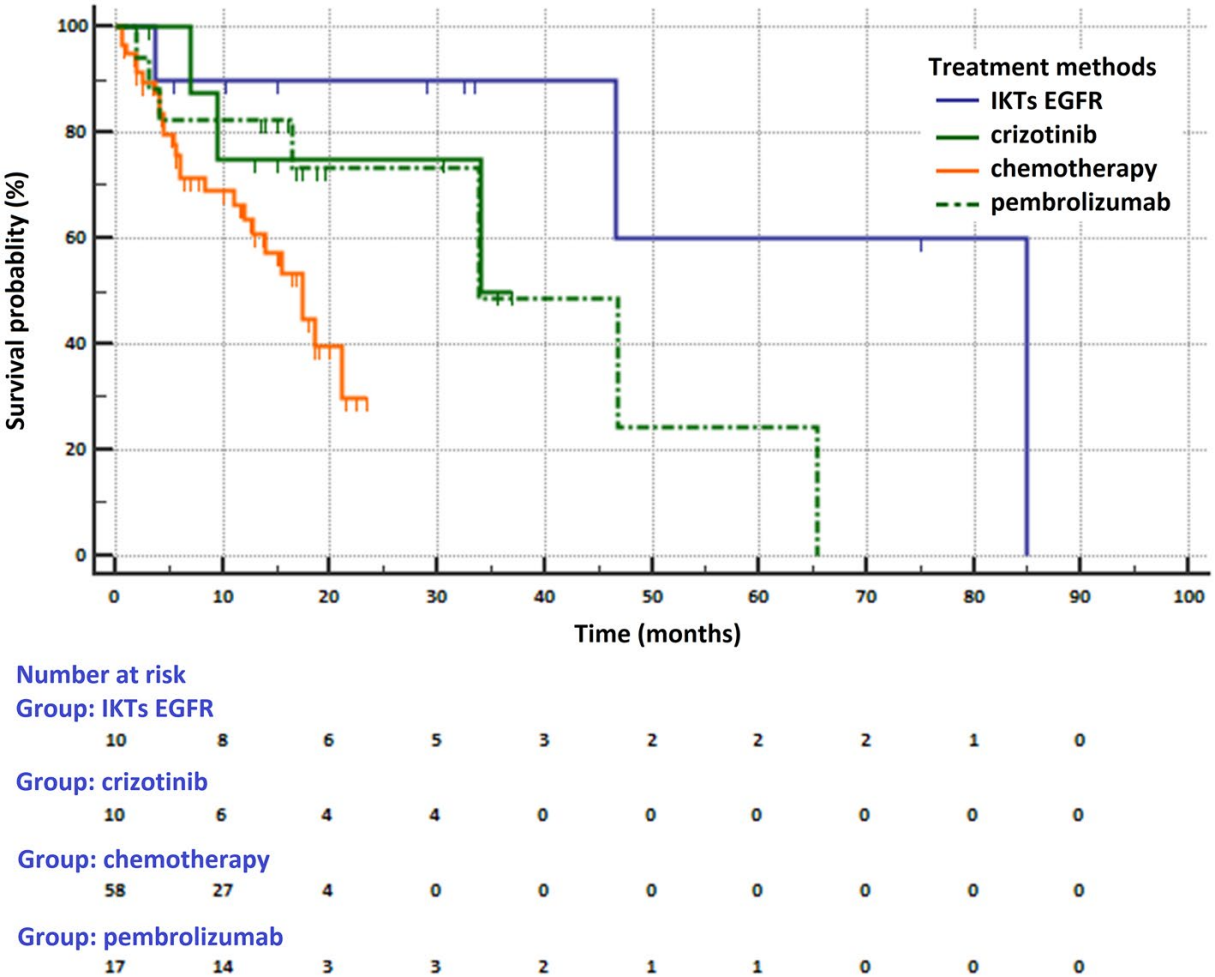
Survival of NSCLC patients treated with various methods (MedCalc Statistical Software version 18.11.6, https://www.medcalc.org).

Median OS in patients receiving various types of first-line treatment were as follows: 34 months (95% CI 9.5–34 months) in crizotinib treated patients, 85 months (95% CI 46.7 months—not reached) in patients who received EGFR TKIs, 33.9 months (95% CI 9–65 months) in pembrolizumab treated patients and 17.5 months (95% CI 12–21 months) in patients who received chemotherapy (p = 0.0136, Fig. 3). The risk of death was lower in patients treated with personalized treatment compared to patients receiving chemotherapy (HR = 0.249, 95% CI 0.0.107–0.578 for patients treated with TKIs EGFR, HR = 0.441, 95% CI 0.156–1.246 for patients treated with crizotinib and HR = 0.532, 95% CI 0.235–1.206 for patients treated with pembrolizumab). Treatment with TKIs EGFR reduced the risk of death compared to treatment with crizotinib (HR = 0.564, 95% CI 0.178–1.784) or pembrolizumab (HR = 0.467, 95% CI 0.1795–1.2175). In contrast, the effectiveness in reducing the risk of death of crizotinib was only slightly greater than that of pembrolizumab (HR = 0.8288, 95% CI 0.267–2.576).

## Material and methods

### Patients

95 NSCLC patients (median age: 65 ± 8.1 years, 39 women, 56 men) diagnosed and treated in Department of Pneumonology, Oncology and Allergology were retrospectively enrolled to the study. Adenocarcinoma was diagnosed in 88 patients and NSCLC NOS (not otherwise specified)—in 7 patients. Patients with *ALK* and *ROS1* rearrangement were diagnosed from January 2016 to December 2017 in our Clinic. We randomly selected patients receiving TKIs EGFR, pembrolizumab or chemotherapy from group of patients with complete clinical information. All enrolled patients had diagnosis of abnormalities in *EGFR* gene (real-time PCR method), *ALK* gene (IHC, FISH and RT-PCR methods), *ROS1* gene (FISH and RT-PCR methods), *MET* gene (RT-PCR method) and *RET* gene (RT-PCR method) as well as assessment of PD-L1 protein expression (IHC method). We enrolled 9 patients with *ROS1* gene rearrangements and 23 patients with *ALK* gene rearrangements as well as 5 patients with exon 14 *MET* gene skipping mutations and one patient with *RET* gene rearrangement. 23 patients had PD-L1 expression on ≥ 50% of tumor cells. Three *ROS1*-positive patients, seven *ALK*-positive patients, and no *MET*-positive patients received crizotinib (problems with drug reimbursement and availability in Poland). 10 patients with *EGFR* gene mutations (6 patients with exon 19 deletions and 4 patients with Leu858Arg substitution in exon 21, all with adenocarcinoma in the IIIB or IV stage, mostly female (7)) received TKIs EGFR, 17 patients with PD-L1 expression on ≥ 50% of tumor cells were treated with pembrolizumab and other patients received platinum-based chemotherapy. These therapies were used in the 1st line of treatment. Demographic and clinic-pathological characteristics of the patients were presented in Table 3.

**Table 3:** Patients characteristics (SD—standard deviation, AC—adenocarcinoma, NSCLC NOS—non-small cell lung cancer not otherwise specified, CTH—chemotherapy).

	Patients with *ROS1* rearrangement	Patients with *ALK* rearrangement	Patients with *EGFR* gene mutations	Patients with *MET* skipping mutation	Patients with PD-L1 expression on ≥ 50% TC	Patients without detected alterations
Number	9	23	10	5	23	25
Age (median ± SD)	66 ± 6 years	69.5 ± 3.5 years	67 ± 10.3 years	69 ± 8.5 years	58.5 ± 4.5 years	65 ± 8.1 years
Gender (number)	Female (4)	Female (11)	Female (7)	Female (3)	Female (10)	Female (7)
	Male (5)	Male (12)	Male (3)	Male (2)	Male (13)	Male (18)
Smoking status	Non-smokers (5)	Non-smokers (13)	Non-smokers (8)	Non-smokers (2)	Non-smokers (4)	Non-smokers (5)
	Smokers (4)	Smokers (10)	Smokers (2)	Smokers (3)	Smokers (19)	Smokers (20)
Pathomorfological diagnosis (number)	AC (9)	AC (19)	AC (10)	AC (4)	AC (20)	AC (25)
		NSCLC NOS (4)		NSCLC NOS (1)	NSCLC NOS (3)	
1st line treatment (number)	Krizotinib (3)	Krizotinib (7)	Gefitinib (1)	CTH (5)	Pembrolizumab (17)	CTH (30)
	CTH (6)	CTH (16)	Erlotinib (5)		CTH (6)	
			Afatinib (4)			

Informed consent has been obtained for all patients. All methods were performed in accordance with the relevant guidelines and regulations. Any aspect of the work covered in this manuscript was approved by the Ethics Committee of the Medical University of Lublin, Poland (No. KE-0254/169/2014).

### Real-time PCR analysis of EGFR gene mutations

DNA was isolated from formalin-fixed paraffin-embedded (FFPE) tumor tissues or from cytological specimens (cell-blocks). DNA was extracted using QIAamp DNA FFPE Tissue Kit (CE-IVD marked, Qiagen, Germany). Isolation was performed according to the manufacturer’s instructions. Concentration and quality of isolated DNA was estimated by spectrophotometry^9^.

Mutations of *EGFR* gene were identified using the EntroGen *EGFR* Mutations Analysis Kit (CE-IVD marked, EntroGen, Woodland Hills, Canada) on Cobas Z 480 real-time PCR system (Roche Diagnostics, USA). We examined all the most common mutations in exons 18 to 21.

### Immunohistochemistry test of abnormal ALK protein and PD-L1 protein expression

Abnormal ALK protein and PD-L1 protein expression was examined using immunohistochemistry test^10,11^. Analyses were performed on 3 µm sections of paraffin-embedded tissue, fixed on Thermo Scientific Superfrost Plus glass slides.

ALK protein IHC staining was conducted on Ventana Benchmark GX platform, using CE-IVD approved anti-ALK Rabbit Monoclonal Primary Antibody (clone D5F3). OptiView Amplification Kit and OptiView DAB IHC Detection Kit were used as a detection system. Hematoxylin counterstaining was incorporated in the staining protocol. As a negative control, Rabbit monoclonal negative control immunoglobulin was used (Ventana Medical System, Tuscon, USA).

CE-IVD approved Ventana SP263 antibody was used for PD-L1 protein IHC staining. The procedure was carried out on Ventana Benchmark GX equipment. OptiView Amplification Kit and OptiView DAB IHC Detection Kit were used as a detection system. Counterstaining using haematoxylin was included in the staining protocol. Rabbit monoclonal negative control immunoglobulin (Ventana Medical System, Tucson, AZ, USA) was used as a negative control.

The slides were assessed by pathologists using an Olympus BX41 microscope.

### Fluorescence in situ hybridization (FISH) analysis of ALK and ROS1 genes rearrangements

All positive results of ALK expression obtained in IHC staining were re-evaluated by FISH method to visualize the presence of *ALK* rearrangement using the Vysis *ALK* Break Apart FISH Probe Kit (Abbot Molecular, USA), paraffin-pretreatment IV and Post-Hybridization Wash Buffer Kit (Abbot Molecular, USA) in fluorescent microscope Axio Scope (Zeiss, Germany)^12^.

In the diagnosis of *ROS1* gene rearrangement, we used the ZytoLight SPEC ROS1 DualColor Break Apart Probe (ZytoVision, Germany), the Vysis Paraffin Pretreatment and Post-hybridization Wash Buffer Kit (Abbott, USA). Fluorescence signals have been assessed using an Axio Scope microscope (Zeiss, Germany)^13^.

The localization and content of tumor cells were examined with H&E staining in serially prepared slides. Interpretation of FISH results was conducted in accordance to American Food and Drug Administration (FDA) and IASLC (International Association for the Study of Lung Cancer) guidelines.

### Reverse transcriptase PCR analysis of ALK, ROS1, RET genes rearrangements and MET gene skipping mutations

Total RNA was extracted from FFPE tissues with the miRNeasy FFPE Kit (Qiagen Inc., Germany) according to the manufacturers’ instructions. RNA concentration was measured with Qubit 4 fluorometers (Invitrogen, Thermo Fisher Scientific, Waltham, USA). RNA samples were stored at − 80 °C until RT-qPCR (reverse transcriptase-quantitative PCR) was performed.

To detect *ALK*, *ROS1* and *RET* gene fusions, as well as *MET* exon 14 skipping mutations we used the Lung Cancer RNA Panel kit (EntroGen, Woodland Hills, Canada) according to the manufacturer’s instruction. There were 8 reactions of twenty microliters in volume of one-step RT-qPCR for one patients. Every reaction mixture contained: 10 µl of One-Step RT-qPCR Reaction Mix, 1 µl of RT Enzyme Mix, 4 µl of Reaction Detection Primer Mix (one from eight) and 5 µl of RNA (concentration 16 ng/µl). RT-qPCR reaction was performed on Illumina Eco real-time PCR platform (Illumina, San Diego, USA) in conditions as follows: 55 °C for 10 min, 95 °C for 1 min and next 40 cycles: 95 °C for 10 s and 60 °C for 45 s. Ct values were obtained and analysis was performed according to the manufacturer’s instruction.

### Statistical analysis

All statistical analyses were performed using MedCalc Statistical Software version 18.11.6 (MedCalc Software, Ostend, Belgium). Data were expressed as a percentage (for the categorized variable), median and standard deviation (for continuous variables). We considered p values below 0.05 to be statistically significant. The analysis of survival was carried out using the Kaplan–Meier estimation method with calculation of the hazard ratio (HR) and 95% confidence interval (CI).

### Ethics approval and consent to participate

The authors declare that accepted principles of ethical and professional conduct have been followed.

## Discussion

The real-time PCR technique is useful, easily feasible and readily available in the diagnosis of genetic alterations in cancer diseases. In NSCLC patients, it is especially useful in the diagnosis of mutations in the *EGFR* gene. However, this method in the diagnosis of gene rearrangements has many limitations. First of all, real-time RT-qPCR requires the use of a reverse transcription step to transcribe mRNA into cDNA. The success of this step depends on the integrity of the mRNA, which may be compromised by the fixation of the biopsy material. Second, not all fusion variants that have recently been described, can be detected by RT-qPCR. Three major variants (v1: E13;A20, v2: E20;A20, and v3: E6;A20) account for more than 90% of lung cancers patients with fusion of *ALK* and *EML-4* (Echinoderm Microtubule-Associated Protein-Like 4) genes. However, RT-qPCR technique may not detect irregular variants and cannot detect *ALK* fusions with other partner sets such as *KIF5B* (Kinesin Family Member 5B) and *KLC1* (Kinesin Light Chain 1). Therefore, the risk of false-negative results and a high failure rate for RNA-based assays on FFPE samples cannot be neglected. In our study, we also found a low sensitivity of the RT-qPCR technique in the diagnosis of *ALK* and *ROS1* genes rearrangements compared to the FISH and IHC methods. What is worse, we also found false-positive results in RT-PCR technique, which was probably associated with errors in mRNA transcription into cDNA. False positive results could also appear in the presence of a unique clone of tumor cells with rearrangements (in the FISH method, the finding of 15% of tumor cells with rearrangements of the *ALK* or *ROS1* gene is considered a positive result)^3^.

Early studies proved that the sensitivity and specificity of RT-PCR for detection of *ALK* gene rearrangements compared with IHC and FISH are good, ranging from 94 to 100%. However, in Soda et al. study, 108 (12%) of 916 specimens were excluded because mRNA was in poor quality^3,14,15^. Cao B et al., using FISH as a standard method, determined the sensitivity and specificity of RT-PCR method at the level of 100% in the diagnosis of selected fusion variants of the *ROS1* gene. Later studies did not show such high sensitivity of the RT-PCR method in the diagnosis of *ALK* gene rearrangements^16^. Lantuejoul et al. showed that 39% (50 of 127) of the cases that were positive by FISH and IHC methods were negative with RT-PCR method^17^. Letovanec et al. reported that RT-PCR provided results for 77 of 96 (80.2%) samples. Using IHC and FISH as a standard method for diagnosis of *ALK* rearrangements, they determined the sensitivity of RT-PCR method at the level of 70.0% and specificity at the level of 87.1%^18^.

The median PFS in our patients with ALK or ROS1 genes rearrangements treated with crizotinib was higher compared to patients from PROFILE 1014 study (23 months (95% CI 6–23) vs. 10.9 months (95% CI 8.3–13.9). However, this data may be due to the weakness of our study. Crizotinib was administered to a small group of patients (10 vs. 174 patients in PROFILE 1014 study). In addition, our group included 4 patients in whom we did not have complete data on the time of disease progression (censored data). They were still treated, or we lost contact with them^19^.

Our observations on the effect on overall survival of chemotherapy or crizotinib used in first-line treatment in NSCLC patients with rearrangements in the *ALK* and *ROS1* genes are difficult to verify based on the results of clinical trials. Median follow-up for OS analysis in PROFILE 1014 study was approximately 46 months. However, in the chemotherapy arm, 84.2% patients received crizotinib in subsequent lines. Moreover, large group of crizotinib-treated patients received a subsequent treatment with higher generation of ALK inhibitors. In our study, there were no patients subjected to crossover or patients who have received subsequent line treatment with ALK inhibitors (lack of reimbursement). Nevertheless, the median OS in our patients treated with crizotinib was 34 months. In PROFILE 1014 study, median OS was not reached in crizotinib-treated patients (95% CI 45.8 months to NR) and 47.5 months in patients who received chemotherapy (95% CI 32.2 months to NR, HR = 0.76, p = 0.0978). After crossover adjustment, there was an improvement in OS that favored crizotinib over chemotherapy (HR = 0.346; 95% CI 0.081–0.718)^19^. In PROFILE 1001 study, median OS of patients with *ROS1* rearrangements treated with crizotinib was 51.4 months ^7^.

We obtained a surprisingly high median OS (85 months) in NSCLC patients with *EGFR* gene mutations. It is caused by a small number of patients with *EGFR* gene mutations enrolled to the study (although the qualification was random) and the use of osimertinib in two patients in the second-line treatment. In clinical trials, patients who used first or second generation TKIs EGFR in the first-line treatment achieved median OS from 22.8 to 34.8 months^1^. However, patients treated with first or second generation of TKIs EGFR in first-line treatment and with osimertinb in second-line treatment in AURA 3 clinical trial achieved a median OS of greater than 40 months (from initiation of first-line therapy)^20^. Median OS in patients with PD-L1 expression on ≥ 50% of tumor cells treated with pembrolizumab in first-line treatment in clinical study KEYNOE-024 after 5 years of follow-up was 26.3 months (33.9 months in our study)^21^.

The leading study for discussion is observation of Kris and co-workers. Authors examined tumors from 1007 NSCLC patients for detection of *KRAS*, *EGFR*, *ALK*, *HER2*, *BRAF*, *PIK3CA*, *MET*, *NRAS*, *MEK1*, and *AKT1* genes abnormalities. Results were used to select a molecularly targeted therapy in 275 of 1007 patients. The median survival was 3.5 years for the 260 patients with an oncogenic driver alterations who received molecularly targeted therapies compared with 2.4 years for the 318 patients with driver mutations or rearrangements who did not receive such therapies (HR = 0.69 p = 0.006). Patients without genetic abnormalities, most frequently treated with chemotherapy, achieved a median overall survival of 2.1 years, which did not differ significantly from the median OS in patients with genetic alterations who could not receive molecularly targeted therapies. The authors concluded that the possibility of extending the survival of patients with molecular driver alterations is largely dependent on the type of treatment, and not on clinical or demographic characteristics, such as gender, age or stage of disease^4^.

The results of the Kris and colleagues are fully consistent with our observations. We showed that patients with genetic abnormalities treated with chemotherapy have an even worse prognosis than patients without these alterations receiving chemotherapy. This is probably due to the fact that patients with *ALK* and *ROS1* genes rearrangements treated with chemotherapy showed metastases to the CNS, which were a very poor prognostic factor. All these observations prove the need for fast and accurate diagnosis of predictive factors in NSCLC patients. Proper treatment could extend the life of advanced NSCLC patients even up to several years and improve their quality of life. Nowadays, this becomes particularly important, when drugs targeting new molecular targets and new generations of drugs targeting EGFR, ALK or ROS1 receptors are being developed.

## Data Availability

The datasets generated during and/or analysed during the current study are available from the corresponding author on reasonable request.
